# MHC-correlated odour preferences in humans and the use of oral contraceptives

**DOI:** 10.1098/rspb.2008.0825

**Published:** 2008-08-12

**Authors:** S. Craig Roberts, L. Morris Gosling, Vaughan Carter, Marion Petrie

**Affiliations:** 1 Evolutionary Biology Group, SCMS, University of NewcastleNewcastle-upon-Tyne NE2 4HH, UK; 2 National Blood ServiceHolland Drive, Newcastle-upon-Tyne NE2 4NQ, UK

**Keywords:** human leukocyte antigen, smell, mate choice, heterozygosity, olfaction

## Abstract

Previous studies in animals and humans show that genes in the major histocompatibility complex (MHC) influence individual odours and that females often prefer odour of MHC-dissimilar males, perhaps to increase offspring heterozygosity or reduce inbreeding. Women using oral hormonal contraceptives have been reported to have the opposite preference, raising the possibility that oral contraceptives alter female preference towards MHC similarity, with possible fertility costs. Here we test directly whether contraceptive pill use alters odour preferences using a longitudinal design in which women were tested before and after initiating pill use; a control group of non-users were tested with a comparable interval between test sessions. In contrast to some previous studies, there was no significant difference in ratings between odours of MHC-dissimilar and MHC-similar men among women during the follicular cycle phase. However, single women preferred odours of MHC-similar men, while women in relationships preferred odours of MHC-dissimilar men, a result consistent with studies in other species, suggesting that paired females may seek to improve offspring quality through extra-pair partnerships. Across tests, we found a significant preference shift towards MHC similarity associated with pill use, which was not evident in the control group. If odour plays a role in human mate choice, our results suggest that contraceptive pill use could disrupt disassortative mate preferences.

## 1. Introduction

Olfaction is important in both human and animal mate choice (e.g. Gosling & Roberts 2001; Havlicek *et al*. 2008). One kind of information available from individual odours is an individual's genotype at the major histocompatibility complex, MHC. Since Yamazaki *et al*.'s (1976) discovery that mice prefer to mate with individuals of different MHC-congenic strains, and that this preference is mediated by chemosensory urinary cues (Yamazaki *et al*. 1979), MHC-associated and apparently odour-mediated mating preferences have been demonstrated in several vertebrate taxa, including fish (Olsén *et al*. 1998), reptiles (Olsson *et al*. 2003) and birds (Freeman-Gallant *et al*. 2003). Mate preference for MHC-dissimilar individuals can be adaptive as it would increase offspring MHC heterozygosity, with beneficial influences on offspring viability through increased resistance to infectious disease or avoidance of inbreeding effects (Potts & Wakeland 1993; Milinski 2006).

MHC-correlated odour preferences have also been demonstrated in humans. In a remarkable study, Wedekind *et al*. (1995) presented male axillary odours, collected on t-shirts worn overnight, to female sniffers. Normally cycling women tested during the follicular phase of their menstrual cycles rated odours of MHC-dissimilar men as more pleasant than odours of MHC-similar men (see also Wedekind & Füri 1997). Additionally, odours of MHC-dissimilar men more often reminded women of current or previous partners, indicating that odour plays a role in partner choice. Subsequent studies on women's preferences have found somewhat mixed results, ranging from similarly disassortative preferences (Santos *et al*. 2005) or a preference for an intermediate level of dissimilarity (Jacob *et al*. 2002) to a null effect (Thornhill *et al*. 2003). Studies of allele sharing in established partnerships also provide mixed results, with one finding disassortative mating (Ober *et al*. 1997), two finding no effect (Hedrick & Black 1997; Ihara *et al*. 2000) and one reporting assortative mating (Rosenberg *et al*. 1983), although this result may be confounded by ethnicity (Roberts & Little 2008). Among real couples, self-reports suggest that women who share fewer alleles with their partner are more content in their relationship and less likely to seek extra-pair partnerships (Garver-Apgar *et al*. 2006). There is thus mixed support for a role of MHC in human partner choice and relationship stability (Roberts & Little 2008).

Against this background, an ancillary result reported by Wedekind *et al*. (1995) deserves further investigation. In contrast to MHC-dissimilar preferences in normally cycling women, pill users preferred odours of MHC-similar men, indicating that pill use might disrupt adaptive preference for dissimilarity. Wedekind *et al*. speculated that this reflected a hormonally induced shift owing to the pregnancy-mimicking effect of the pill, leading to increased association with kin who could assist in childcare. However, this shift could be costly if it results in choice of more MHC-similar partners: MHC similarity in couples may lead to increased risk of recurrent spontaneous abortion and longer interbirth intervals (review in Beydoun & Saftlas 2005), and perhaps ultimately to partnership breakdown if odour perception plays a part in maintaining attraction to partners (Vollrath & Milinski 1995).

Here, we specifically test the possibility that pill use alters female preferences for male body odour. Wedekind *et al*.'s (1995) paper has been criticized (Hedrick & Loeschke 1996) on the grounds that the pill-using group was relatively small (18, compared with 31 non-users). Furthermore, because it was between-subjects in design, differences in MHC-correlated preferences could be due to underlying differences between pill-using and non-using women. To address the second point in particular, we used a within-subjects experimental design, comparing preferences before and after initiating pill use. Our design was based as far as possible on Wedekind *et al*.'s (1995) study, in which women rated odours of six men (three MHC similar and three MHC dissimilar), but each woman was tested twice, with an approximately 3-month interval. In session 1, women were all tested in the late follicular phase of their cycle; our aim was that approximately half would initiate pill use shortly afterwards, while half would continue to cycle as normal.

## 2. Material and methods

### (a) Participants

Most female participants were students or staff at Newcastle University, recruited by advertisement or word of mouth; a small number were recruited from local contraceptive clinics. They were offered £25 in compensation for time, travel and inconvenience. Participation requirements included not using any form of hormonal contraception, including the Depo-Provera injection, either currently or within the preceding three months, not being pregnant, experiencing regular cycles and being heterosexual. Women included in the pill group were either planning or considering to use the pill, and were willing to schedule initiation around the experiment: for ethical reasons, allocation to the pill/control group was entirely the decision of the volunteers, not the experimenters.

We registered 193 women, aged 18–35, as participants, of whom 97 completed the experiment (attended both sessions). We included some additional women in analyses based on either the first or second sessions, and we excluded some in certain analyses. Total sample sizes were 110 for session 1 (none using the pill), 100 for session 2 (60 non-users, 40 pill users) and 97 for the within-subjects comparisons across sessions (60 in control group, 37 in pill group; full details in the electronic supplementary material, table S1).

Male participants were 97 heterosexual, non-smoking students or staff, aged 18–35, paid £10 per odour donation.

### (b) Genotyping

We collected venous blood in 6 ml EDTA-lined vacuettes. Samples were genotyped by polymerase-chain reaction using sequence-specific primers (PCR-SSP), at human leukocyte antigen-A (*HLA-A*), -*B* and -*DRB1* loci, in the National Blood Service Tissue Typing laboratory in Newcastle-upon-Tyne. At *HLA-A*, 15 different alleles were recorded and individuals were homozygous in 15/110 women and 11/97 men (*HLA-B*: 32, 10/110, 5/97; *HLA-DRB1*: 17, 15/110, 10/97). Allele frequencies are shown in the electronic supplementary material, table S2.

### (c) Odour collection

Male participants (odour donors) were supplied with a white cotton t-shirt (pre-washed using unperfumed detergent) in a resealable plastic bag. Shirts were worn in bed for two consecutive nights, returned to their bags each morning, and delivered to the experimenters on the second morning. On delivery, shirts were cut in half (from navel to throat) and frozen at −30°C until use (they were discarded if unused within 3 months). Men wore several shirts; on each occasion, they were instructed to (i) refrain from using perfumed products on either day preceding t-shirt use, (ii) instead use a non-perfumed soap (Simple, which we supplied), (iii) avoid heavy drinking and smoky bars, (iv) avoid spicy foods, (v) refrain from sexual activity, and (vi) sleep alone. We could not verify whether donors followed the instructions, but we asked women to note shirts that smelled of deodorant/detergent or tobacco smoke. Analyses were carried out including these shirts and also omitting them.

### (d) Procedure

For each woman, we pre-selected three MHC-similar and three MHC-dissimilar men. On average, women shared 3.20 alleles with the MHC-similar men (range=2.0–4.67, s.d.=0.66), and 0.02 alleles with MHC-dissimilar men (0–0.67, 0.09). These means compare favourably with Wedekind's experiment (similar 3.3, dissimilar 0.1; Wedekind *et al*. 1995).

At the start of their next menstrual cycle, each woman's first test (session 1) was scheduled; following Wedekind *et al*. (1995), this was between days 10 and 14 of their cycle (where possible, on day 12). Half of one t-shirt from each donor was removed from the freezer 2 hours before the test, placed in a clean glass jar (labelled 1–6), sealed with an aluminium foil lid and left at room temperature. Order of odours was alternated (e.g. MHC-similar odours in jars 1, 3 and 5) and balanced across participants. Immediately before smelling, jars were shaken thoroughly, inverted several times and a triangular ‘nose-hole’ was cut in the lid. Women were instructed to first smell each jar briefly, assess variability in the odours and then begin rating shirts in order. Women took as long as they wished and were left alone in the room. Ratings used a 7-point Likert scale, on three measures. The first two (odour pleasantness, odour intensity) were as used by Wedekind *et al*. (1995). The third (odour desirability) was phrased as follows: ‘Based on this smell, how much would you like this man as a long-term partner?’ The scale was anchored by the phrases ‘Not at all’ and ‘Very much’. We included this question in view of Wedekind *et al*.'s (1995) suggestion to explore other contexts and because use of long-term relationship contexts in judgements influences ratings in facial judgements (e.g. Little *et al*. 2002). We considered also asking for a rating of ‘sexiness’, but this has been shown to correlate highly with pleasantness ratings (Wedekind *et al*. 1995; Thornhill *et al*. 2003). Women were asked to note if shirts reminded them of (i) deodorant, (ii) tobacco smoke, (iii) a partner or former partner, and (iv) a relative. Shirts were discarded after use. Following smelling, women completed a background questionnaire (e.g. current relationship status, self-rated attractiveness).

Women re-contacted the researchers at the beginning of the third cycle after session 1, and a second appointment was made. The median number of days between sessions was 95. Session 2 followed the same procedure, except that the order of presentation was altered (participants were informed of this). Women in the pill group were tested on days 5–9 of their pill packet (corresponding to days 10–14 since the first day of bleeding when using the combined pill).

In addition to these ratings, 86 women (controls: 58, pill group: 28) repeated odour ratings approximately 1 hour later, to investigate rating repeatability. Jars were reordered beforehand according to a predefined random order. In the interim, women undertook some other non-smelling tasks (e.g. rating faces: Roberts *et al*. 2005*b*,*c*).

### (e) Effect of freezing

Women who had completed the main smelling tests were, if shirts were available, given this experiment as an additional task. Four shirts from the same man were presented, where one was fresh, one had been frozen for one month, one for two months and one for three months (in the latter three cases: ±1 week). The four shirts were only presented to one woman, in random order with respect to storage period. Women (*n*=42, all in fertile phase) rated shirts as before and then ranked them in the order of preference.

**Figure 1:**
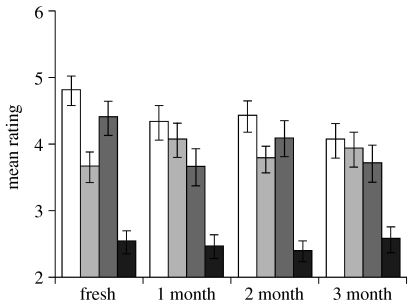
Mean (±s.e.) scores for odour pleasantness (white bars), intensity (light grey bars) and desirability (dark grey bars) and mean preference rank (black bars) according to the length of frozen storage (*n*=42).

There was no significant effect of freezing on any of the four measures (figure 1): pleasantness (ANOVA: *F*_3,167_=1.58, *p*=0.197), intensity (*F*_3,167_=0.54, *p*=0.659), desirability (*F*_3,167_=1.63, *p*=0.186) or preference rank (*F*_3,167_=0.19, *p*=0.905). Figure 1 suggests the possibility of a slight decrease in pleasantness and desirability between fresh and frozen samples, but no consistent effect of length of time in frozen storage.

### (f) Analysis

For consistency with Wedekind *et al*. (1995), we analyse mean scores given to three MHC-similar and MHC-dissimilar men within either session 1 or 2 using paired *t*-tests, using both women and men as units of analysis. The latter is potentially more powerful (Wedekind *et al*. 1995) because it controls for cues unrelated to MHC, leaving dissimilarity of raters as the only variable. To test for differences across sessions, we used doubly multivariate repeated-measures ANOVA (Tabachnick & Fidell 1996), with group (pill, non-pill) as the between-subjects factor, and both session and rating (odour pleasantness, intensity and desirability) as the two within-subject measures. Difference scores were approximately normally distributed (Kolmogorov–Smirnov tests, all *p*>0.05).

Initial analyses included all women and shirts. Subsequent analyses used a core sample that included participants who were white and of British origin, to avoid confounding variables and minimize potential effects of population stratification in allelic frequencies (Roberts *et al*. 2005*b*), and excluded shirts that reminded smellers of tobacco or perfumed products. One woman failed to record one partner desirability score, and another omitted one intensity rating.

During session 2, we recorded the brand of pill used (where appropriate). All but two women used a combined monophasic brand, including Microgynon (25), Cilest (3), Dianette (3), Yasmin (2), Eugynon (1), Femodene (1), Femodette (1) and Ovranette (1). One used Trinodial (a phasic pill) and another used Femulen (a progestogen-only pill, POP). In the pill analyses reported, these two women were excluded (in the core sample, this exclusion applied only to the woman using Femulen).

Odour associations with the remembered odours of current or ex-partners and odours of relatives were analysed using Fisher's exact tests, following Wedekind *et al*. (1995).

To investigate the repeatability of ratings, Spearman rank correlation coefficients were calculated for scores awarded to the six shirts by individual raters, either within sessions (interval approx. 1 hour) or between sessions (interval approx. 95 days); distributions of these coefficients were tested against chance (zero) using one-sample *t*-tests.

## 3. Results

### (a) Correlations between dimensions

Odour pleasantness was strongly correlated with ratings of partner desirability (*r*_s_=0.854, *n*=659, *p*<0.001). Both pleasantness and desirability were equally and negatively related to perceived odour intensity (session 1, *r*_s_=−0.325, *n*=659 and 658, *p*<0.001).

### (b) Repeatability

Within-session correlations between pleasantness ratings were highly skewed and more positive than expected by chance (women not using the pill: *t*_57_=4.82, *p*<0.001; pill users in session 2: *t*_27_=3.62, *p*=0.001; see the electronic supplementary material, figure S1). Between-session ratings were also correlated in the control group (*t*_59_=4.86, *p*<0.001), but correlations were no higher than expected by chance for pill users (*t*_36_=0.66, *p*=0.515). Between-session repeatability in pleasantness ratings was significantly higher in the control group than the pill group (Wilcoxon test, *z*=2.26, *p*=0.024).

Similar patterns were found for odour intensity and desirability (electronic supplementary material, figure S2). However, between-session ratings of odour intensity by the pill group were highly correlated (*t*_36_=3.02, *p*=0.005), indicating that low repeatability was specific to ratings that indicate odour preference (pleasantness, desirability) rather than to changes in women's olfactory sensitivity.

### (c) Preferences in normally cycling and pill-using women

To our surprise, we found no significant effect of MHC dissimilarity on odour pleasantness or desirability scores in session 1, where no women were using the pill (*p*>0.68, table 1). Intensity ratings of all 110 women showed a tendency for odours of dissimilar men to be rated as stronger, but this non-significant effect was weakened when analysis was restricted to the core sample of British women and shirts not perceived as smelling of tobacco or perfumed products. Across all ratings, there was no correlation between allele sharing and either odour pleasantness (*r*_s_=−0.002, *n*=660, *p*=0.95), partner desirability (*r*_s_=0.013, *n*=659, *p*=0.73) or intensity (*r*_s_=−0.046, *n*=659, *p*=0.24).

In session 2, where some women were using the pill, we again found no significant differences in any comparison (electronic supplementary material, table S3). Our results therefore suggest that, at least in our sample, there was neither a significant general preference for MHC dissimilarity across normally cycling women, nor a significant preference for MHC similarity associated with pill use.

We checked whether the non-significant effect described above might be owing to the inclusion of a proportion of men who, across the sample, were assessed only under one condition (i.e. only as a MHC-similar/dissimilar man). This might be a problem if the odours of such men were unusual or especially (un)attractive. Of all shirts rated in this experiment, 47 were from men assessed only as MHC similar, and 48 from men assessed only as MHC dissimilar (7% each; the other 86% of shirts were from men assessed by at least one woman in both MHC-similar and MHC-dissimilar conditions). There were no differences in odour pleasantness, desirability or intensity between these men (independent-samples *t*-tests, all *p*>0.17). Furthermore, recalculating mean MHC-similar and MHC-dissimilar ratings for each woman, with these men excluded, had little effect on the results (compared with table 1: pleasantness, *t*_109_=0.32, *p*=0.75; desirability, *t*_109_=0.24, *p*=0.81; intensity, *t*_109_=1.98, *p*=0.051; core sample: all *p*>0.4).

Following previous studies (Wedekind *et al*. 1995; Wedekind & Füri 1997), we next compared scores assigned to male shirts when presented to MHC-similar and MHC-dissimilar women (i.e. men as unit of analysis). In this analysis, we used *z* scores (i.e. with a mean of zero and standard deviation of 1) to control for variability in the use of the rating scale by individual women (cf. Roberts *et al*. 2005*b*; full details, also using raw scores, are given in the electronic supplementary material, table S4). In session 1, we found no difference in ratings when individual men's odours were assessed as MHC similar or MHC dissimilar, neither in the entire sample (paired *t*-tests, *n*=79 men, *t*=0.54, 0.35 and 0.93 for pleasantness, intensity and desirability, respectively, all n.s.) nor the core sample (*n*=52, *t*=1.06, 0.38 and 0.27, all n.s.; electronic supplementary material, table S4). We found no effects of male heterozygosity on odour pleasantness, intensity or partnership desirability; mean pleasantness/desirability scores were higher for heterozygotes in non-users, and lower in pill users, but these differences did not approach significance (electronic supplementary material, table S5). Following Wedekind *et al*. (1995), we tested for an association between MHC dissimilarity and the number of times women indicated that shirt odours reminded them of either partners or relatives. However, we found no significant effects in either session (electronic supplementary material, table S6).

### (d) Changes in relation to pill use

Although we detected no general MHC-associated preferences, we next looked for potential shifts in preferences across sessions. We first calculated a within-session difference score between mean ratings of MHC-similar and MHC-dissimilar odours for each rater, subtracting similar scores from those for dissimilar odours (positive scores indicate preference for MHC-dissimilar odours). We then used doubly multivariate repeated measures ANOVA to test for changes in relative preference for MHC dissimilarity.

**Figure 2:**
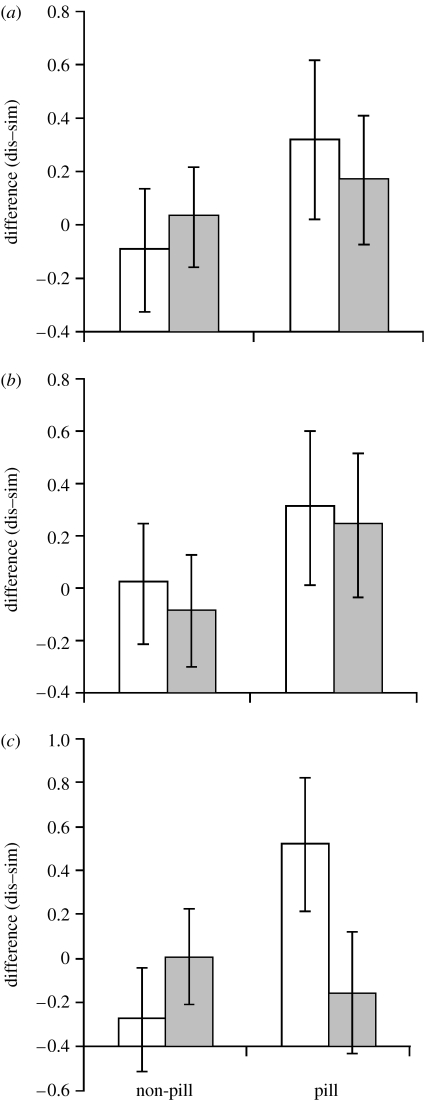
Mean difference in odour ratings for MHC-similar and MHC-dissimilar men by pill-using and non-pill-using women in two rating sessions (open bar, first test; filled bar, second test). Positive scores indicate preference for MHC-dissimilar odours. (*a*) odour pleasantness ratings, (*b*) odour intensity and (*c*) odour desirability.

We found no significant main or interaction effects when using the whole sample. However, with the core sample, we found a significant session–group interaction (*F*_3,71_=3.05, *p*=0.034), driven mainly by desirability ratings (*F*_1,73_=3.63, *p*=0.061; pleasantness *F*_1,73_=0.22, *p*=0.64; intensity *F*_1,73_=0.01, *p*=0.92). Excluding the one woman who used a progesterone-only pill did not affect the main interaction (*F*_3,70_=3.07, *p*=0.034) but increased the effect of desirability ratings (*F*_1,72_=4.19, *p*=0.044). This interaction (figure 2) is indicative of a decreasing preference for dissimilarity across the two sessions among the pill-using group and, to a lesser extent, an increasing preference for dissimilarity in the control group.

Finally, we considered the possibility that differential use of the rating scale between sessions might obscure any relevant effects (e.g. women's familiarity or distaste for the odours may have changed as a result of experience in session 1, and might have differed between the pill and control groups). We therefore repeated the analysis using *z* scores. This made little qualitative difference to the analysis, again showing a significant session–group interaction (*F*_3,64_=2.82, *p*=0.046), driven by desirability ratings (*F*_1,66_=4.07, *p*=0.030; pleasantness *F*_1,66_=0.55, *p*=0.46; intensity *F*_1,66_=0.37, *p*=0.54).

### (e) Differences between women

We detected a difference in the use of rating scales between treatment groups, which was evident even in session 1, before the pill group began pill use: mean scores given to all six shirts were higher for the pill group, for both odour pleasantness (*t*_108_=3.28, *p*=0.001) and partner desirability (*t*_108_=3.21, *p*=0.002). However, there was no difference in the ratings of odour intensity (*t*_108_=1.19, *p*=0.238), indicating that the differences for pleasantness and desirability were unrelated to differences in the ability to smell the odours.

**Figure 3:**
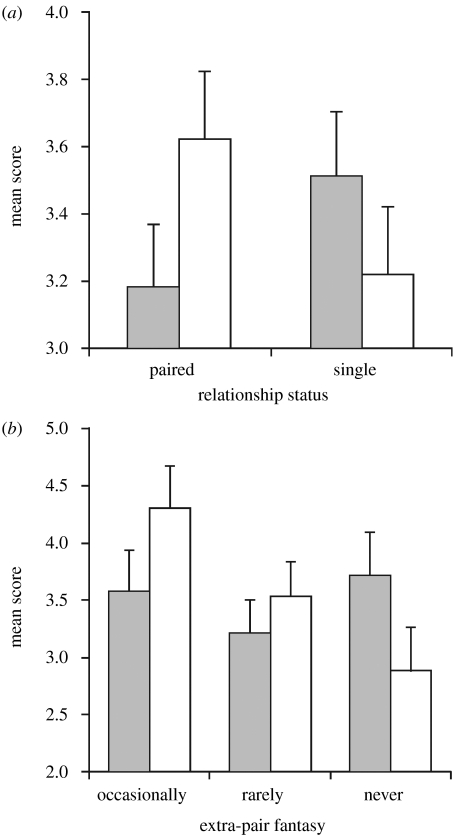
Effects of relationship status on MHC-correlated odour preferences. (*a*) Differences in partnership desirability ratings of MHC-similar and MHC-dissimilar male body odours by 42 single and 43 paired women. The interaction is significant (*p*=0.033). (*b*) Effect of the frequency with which women fantasize about sex with other men (*p*=0.080). Filled bar, MHC-similar male body odour; open bar, MHC-dissimilar male body odour.

We also noted a significant difference in responses from women who were grouped according to whether they reported being in a current relationship. In session 1 (none using the pill), we found a significant relationship status–MHC interaction (*F*_1,83_=4.72, *p*=0.033), such that paired women gave higher partnership desirability scores to MHC-dissimilar men, and single women preferred MHC-similar men (figure 3*a*). The same interaction for odour pleasantness ratings bordered on significance (*F*_1,83_=3.92, *p*=0.051), but there was no effect for odour intensity (*F*_1,83_=1.06, *p*=0.307). Relationship length was unrelated to MHC-odour preference, but among women in relationships, we found a near-significant association between MHC-odour desirability scores (but not pleasantness or intensity) and the frequency with which women reported fantasizing about sexual relationships with other men (*F*_2,45_=2.68, *p*=0.080), such that women who did so more frequently gave higher desirability scores to MHC-dissimilar odours (figure 3*b*).

There was no significant association between intention to initiate pill use and current relationship status: 22/41 pill users and 40/72 non-users reported being in a relationship (*X*^2^=0.45, *p*=0.85). Despite this, in view of the effect of relationship status, we repeated the main repeated-measures analysis of pill use on ratings, this time adding relationship status as a between-subjects factor. The results remained qualitatively unchanged: there was a significant session–group interaction (*F*_3,69_=2.95, *p*=0.039), driven by odour desirability (*F*_1,71_=3.53, *p*=0.064), but no significant interactions for session–relationship status (*F*_3,69_=0.46, *p*=0.712), relationship status–group (*F*_3,69_=0.45, *p*=0.717) or session–relationship status–group (*F*_3,69_=0.04, *p*=0.990).

There was no relationship between self-rated attractiveness of women raters and pill use (Mann–Whitney tests; facial attractiveness: *U*=1254, *p*=0.82; physical attractiveness: *U*=1174, *p*=0.44; both *n*=39_pill_ and 66_non-pill_). Self-rated facial attractiveness did not vary among single or paired women (*U*=1349.5, *p*=0.90, *n*=57_single_ and 48_paired_), but self-assessed physical attractiveness was higher among paired women (*U*=1056.5, *p*=0.038). However, tests of MHC preference in the first test revealed no effect of physical attractiveness (entered as a covariate) on preference either in a model without relationship status (main effect, *p*=0.37; interaction *p*=0.70) or with it (main effect, *p*=0.44; interaction *p*=0.42; the MHC–relationship status interaction remained significant, *F*_1,77_=4.17, *p*=0.045). Including self-rated attractiveness (either facial or physical) as a covariate in the main repeated measures ANOVA across tests only increased the significance of the session–group interaction reported above (*F*_3,66_=4.18, *p*=0.009 and *F*_3,66_=3.45, *p*=0.021, for facial and physical attractiveness, respectively).

## 4. Discussion

Although several studies have reported significant effects of MHC dissimilarity on women's preferences for male body odour, we were unable to replicate this on our main sample of women, in which none were pill users and all were in the follicular cycle phase. We based the design of our study on that of Wedekind *et al*. (1995); like them, (i) we tested preferences among three MHC-similar and three MHC-dissimilar odours, (ii) odours were captured on cotton t-shirts worn in bed, (iii) odour donors were asked to avoid potentially confounding environmental odours, (iv) menstrual cycle stage was controlled and so on.

However, there were nonetheless some methodological differences that could have accounted for the differences in the results. Wedekind *et al*.'s women used nasal sprays to help their sense of smell, and read Su¨skind's novel *Das Parfum* to raise awareness of their smell perception. Ours did neither of these, but these omissions are unlikely to be critical since other studies that omitted these requirements report significant effects of MHC on odour perception (e.g. Santos *et al*. 2005). Although not specified in Wedekind *et al*. (1995), Wedekind & Füri (1997) noted that odour donors had unshaven axillae and that odour collection occurred during the summer. We did not collect information on axillary shaving (but few British men do so) and shirt wearing took place all-year round. We did not use untreated cotton shirts (although shirts were washed with unperfumed detergent) and used halved rather than whole shirts, which may have reduced stimulus intensity. We used glass jars rather than cardboard boxes to present the shirts, and capped these with aluminium rather than plastic foil, because glass can be washed and plastics absorb odours. Finally, we asked women to briefly sniff all shirts before undertaking rating, which Wedekind *et al*. did not, because we felt this would allow women more consistent assessment of the shirts and reduce order effects. While some further differences were introduced owing to our more complex experimental design, most were intended as improvements on design, although it remains possible they may have obscured women's ratings.

A more serious methodological difference was that, while Wedekind *et al*. (also Thornhill *et al*. 2003; Santos *et al*. 2005) presented freshly worn t-shirts to their smellers, we stored shirts in a freezer between collection and presentation. However, Thornhill *et al*. found no significant preference related to MHC, suggesting that a null effect cannot solely be attributed to freezing. Conversely, other odour studies using frozen samples have detected predicted and biologically meaningful effects, such as the relationships between odour and both facial attractiveness and bodily symmetry (Rikowski & Grammer 1999), ovulatory status effects on women's odour attractiveness (Singh & Bronstad 2001), and of particular relevance here, the ability of human smellers to detect genetic relatedness through body odour (Roberts *et al*. 2005*a*) and of a perfumer to describe MHC-associated odours (after freezing for more than 1 year: Wedekind *et al*. 2007). Furthermore, while the predicted positive effect of MHC dissimilarity on ratings was not supported, we did detect other differences associated with pill use and relationship status, and ratings within and between test sessions were highly repeatable. Our supplementary experiment using frozen t-shirts from the same men found no significant effect of freezing over 3 months on odour ratings or preference rank. Thus, though it remains a possibility, we think it unlikely that frozen storage of samples (or other differences) were responsible for the null effect of MHC dissimilarity.

It is also possible that the null effect might have been due to the inclusion of men who were assessed under either the MHC-similar or MHC-dissimilar condition, but not in both. For example, it could have been that those included only as MHC-similar men had especially attractive/weak odour, while those included only as MHC-dissimilar men had very unattractive/intense odours. MHC studies should aspire to balance inclusion in either condition across the sample to avoid these non-MHC related effects (Wedekind 2002). In our study, logistical reasons led to 14 per cent of ratings relating to men who contributed in only one condition. However, we found no evidence for systematic differences in odour pleasantness, desirability or intensity, suggesting that this was unlikely to be responsible for the null effect. Furthermore, the effect of excluding these men's shirts from analyses was to reduce, rather than enhance, the difference between mean similar and mean dissimilar ratings.

Our data provide further evidence that use of oral contraceptives influence women's MHC-correlated odour preferences. The significant session–group interaction, whereby ratings shift in favour of MHC similarity after initiating pill use, in contrast to the control group, is consistent with Wedekind *et al*.'s (1995) suggestion that pill use may disrupt adaptive mate preferences. Indeed, our results are the first to test this suggestion empirically. The slight change in the control group of non pill-users could arguably be interpreted in terms of increased experience in olfactory testing, eliciting a slightly stronger preference for MHC dissimilarity, but this was not matched by the pill group. Although we had only one woman in our pill-using group who used a POP, excluding her from analysis improved the explanatory power of desirability ratings on change in odour preference. We could therefore speculate that POP use may have less influence on this change than the combined pill, but this is based on only one woman and needs further testing.

At least two alternative explanations for the difference among Wedekind *et al*.'s (1995) groups of pill-using and non-using women could be proposed. One is that the association between MHC-similarity preference and pill use is a by-product of increase in preference for MHC heterozygosity, since heterozygous men are on average more likely to share alleles with women raters (cf. Roberts *et al*. 2006) and Thornhill *et al*. (2003) report greater preference for heterozygotes in the luteal phase. However, our results indicated no difference in preferences for heterozygotes in either non-users or pill-users. A second possibility, one which stimulated this study, is that there might be pre-existing behavioural differences between women who choose to use the pill and those who do not. Indeed, we found that pill users used rating scales differently, awarding higher scores, on average, than non-users. Importantly, this difference was apparent even before they initiated pill use, although we do not know the reason for this. While absolutely higher ratings could not lead to the difference found by Wedekind *et al*., it could potentially arise from a positive association between pill use and other attributes such as attractiveness or likelihood of being in a sexual relationship. We found no evidence for an effect of attractiveness on preferences or pill use, but we did find an association between relationship status and MHC preference. However, in direct contrast to what might be inferred from Wedekind *et al*.'s data, paired women showed higher preference for MHC dissimilarity while single women preferred MHC similarity. This intriguing effect of relationship status is discussed further below, but it is worth noting that it may be at least partly responsible for the variability in findings across MHC-odour studies.

Our results therefore cannot, at face value, provide an explanation for the pill effect previously reported, but they do emphasize the way in which current circumstances can modulate preferences based on genetic similarity. Mouse studies suggest that odour preference expression varies depending on reproductive status and behavioural context, since lactating female mice prefer to associate with MHC-similar females, presumably using odour (Manning *et al*. 1992), while females in oestrus prefer odours of MHC-dissimilar males (and other aspects of genetic quality involved in mate choice, Roberts & Gosling 2003). In our study, paired women expressed greater preferences for MHC dissimilarity in odours of unfamiliar men, and there was a non-significant association between fantasizing about extra-pair relationships and MHC-dissimilar odour preference. Such expression of enhanced preference for dissimilarity might be interpreted within the context of desired attributes in extra-pair partners as a means to increase offspring heterozygosity, in common with similar preferences in birds (e.g. Petrie & Kempenaers 1998; Blomqvist *et al*. 2002), although it is curious that the effect was elicited most strongly in the long-term context question—perhaps this question focuses raters more successfully on desired mate choice characteristics than does rating of odour pleasantness. It may also be that paired women can evaluate odours more accurately, and thus discriminate MHC dissimilarity more effectively, because they have more intimate recent experience of male odour (although we do not know why single women should have preferred MHC-similar men). Similarly, women in established partnerships express clearer or different preferences for traits indicating additive genetic variance than single women, in both visual (Little *et al*. 2002) and olfactory (Havlicek *et al*. 2005) modalities, but the extent to which these discrepancies ultimately reflect underlying strategic variation or differences in experience remains a question for further study.

We do not know whether the change in preferences related to pill use is sufficiently strong to influence partner choice, but it could do so if odour plays a significant role in actual human mate choice. Some studies have suggested that women consider the olfactory domain to be an important factor in their assessment of potential partners (e.g. Havlicek *et al*. 2008). Although we were unable to replicate the effect, Wedekind *et al*.'s (1995) demonstration of an association between MHC dissimilarity and the reminiscence of current or previous partners suggests that the influence of MHC-odour cues may extend beyond the laboratory. If this is the case, our results indicate that use of the contraceptive pill could lead to choice of an otherwise less preferred partner.

## Figures and Tables

**Table 1:** Mean scores given to three MHC-similar and three MHC-dissimilar male odours by 110 normally cycling women tested during the late follicular phase (session 1). The core sample excludes non-UK women and shirts worn by non-UK men or those that were noted by the participant as smelling of tobacco smoke or fragranced products.

	mean±s.e.				
				
measure	similar	dissimilar	paired *t*	d.f.	*p*
*all women, all shirts*					
pleasantness	3.95±0.08	3.89±0.09	0.41	109	0.685
desirability	3.47±0.10	3.42±0.11	0.37	109	0.713
intensity	4.25±0.09	4.50±0.09	1.95	109	0.053
*core sample (UK women, no confounds)*					
pleasantness	3.77±0.11	3.87±0.13	0.57	84	0.569
desirability	3.35±0.13	3.42±0.14	0.44	84	0.661
intensity	4.18±0.13	4.32±0.12	0.78	84	0.436

## Supplementary Material

Additional tables and figures Methodological detail, repeatability of ratings and additional results
