# Supplementary material for: MHC-correlated odour preferences in humans and the use of oral contraceptives
Source: Proc Biol Sci. 2008 Aug 12;275(1652):2715–22. doi: 10.1098/rspb.2008.0825 (PMC2605820; doi:10.1098/rspb.2008.0825)

ELECTRONIC SUPPLEMENTARY MATERIAL

Roberts S.C., Gosling L.M., Carter V. & Petrie M. (2008)
MHC-correlated odour preferences in humans and the use of oral contraceptives

Table S1. Female participant sample size. The control group includes women who neither used, intended to use nor actually used oral contraceptives during the study. The Pill group includes women who intended to use oral contraceptives (for Session 1) and/or actually began using them between Sessions 1 and 2. Numbers in brackets denote women who were not from the UK or from a different ethnic origin, who were excluded from certain analyses.

	Control group	Pill group	Total
**Women tested in both sessions**[1]	60 (8)	37 (4)	97
**Women tested in Session 1 only**[2]	12 (4)	1 (1)	13
**Women tested in Session 2 only**[2]	0	3 (1)	3

Footnotes

[1]A total of 193 women, aged 18-35, were registered as study participants, of whom 97 completed all study requirements (visited the lab for two testing sessions approximately 3 months apart; each session lasted approximately 1h and included some additional tasks such as rating faces). Of these, 60 did not use the pill and 37 began using it during the experiment.

[2]In addition to the 97 who completed all aspects of the study, we were also able to include in selected analyses 13 who completed the Session 1 but not Session 2 (of whom 1 expressed intention to begin pill use) and 3 who began using the pill before Session 1 (they were tested only as pill users in the between-subjects comparison of users versus non-users). In summary, total sample sizes were therefore 110 for Session 1 (all non-users), 100 for Session 2 (60 non-users, 40 pill users) and 97 for the within-subjects comparisons (60 in control group, 37 in pill group).

Twelve women were from outside the UK, or from a different ethnic origin, and were thus excluded in some analyses to control for potential differences in allele frequencies between populations (leaving 52 in the control group and 33 in the pill group). 5 of the 13 who completed only Session 1 were from outside the UK (including the one who expressed intention to begin pill use), as was 1 of the 3 women who were tested only as pill users.

Table S2. Relative frequencies of MHC alleles at A, B and DRB1 loci.

HLA Locus	Allele number	Relative frequency (Females)	Relative frequency (Males)
A	1	18.18	15.45
	2	28.64	23.64
	3	14.55	13.64
	11	6.36	4.55
	23	2.73	3.64
	24	10	8.18
	25	2.73	0.45
	26	2.73	3.64
	29	2.73	4.09
	30	1.82	2.73
	31	2.73	1.36
	32	1.36	4.09
	33	0.91	0.45
	68	4.09	2.27
	69	0.45	0
B	7	18.64	11.82
	8	14.09	12.73
	13	3.18	0.91
	14	1.36	1.36
	15	0.45	0
	18	3.64	2.27
	27	3.18	4.09
	35	5.91	5.91
	37	0.91	0.45
	38	3.18	3.18
	39	1.82	1.36
	41	0	0.45
	42	0	0.45
	44	14.09	16.82
	45	0.91	0.45
	47	0.45	0
	49	0.91	1.82
	50	0.91	0.91
	51	4.55	5.00
	52	1.36	0.45
	53	0.45	0
	55	1.82	1.82
	56	0	0.91
	57	3.18	2.27
	58	0	0.45
	60	4.55	4.09
	61	0.45	1.36
	62	6.36	5.45
	64	0	0.91
	65	2.73	0.45
	71	0.45	0
	72	0.45	0
DRB1	1	8.64	7.27
	4	15.00	17.73
	5	0.45	0
	6	0.45	0

	7	15.45	15.45
	8	1.82	1.36
	10	0.45	0
	11	8.64	7.73
	12	1.82	0.45
	13	9.55	9.55
	14	0.45	1.82
	15	20	11.36
	16	0	0.91
	17	13.64	13.18
	52	0	0.45
	53	0.45	0
	103	3.18	0.91

Table S3. Mean scores given to 3 MHC-similar and 3 MHC-dissimilar male odours in Session 2 by either normally-cycling or pill-using women.

Measure	Mean ± s.e.		Paired *t*	*df*	*P*
	Similar	Dissimilar			
Normally cycling women, all shirts					
Pleasantness	3.85 ± 0.11	3.87 ± 0.10	0.12	59	0.909
Desirability	3.51 ± 0.12	3.61 ± 0.12	0.62	59	0.538
Intensity	4.15 ± 0.12	4.30 ± 0.12	0.97	59	0.336
Normally cycling women, core sample					
Pleasantness	3.93± 0.13	3.93 ± 0.14	0.0	48	1.0
Desirability	3.67 ± 0.16	3.68 ± 0.15	0.02	48	0.987
Intensity	4.02 ± 0.16	4.02 ± 0.17	0.03	49	0.975
Pill-using women, all shirts					
Pleasantness	4.11 ± 0.15	4.23 ± 0.12	0.68	39	0.503
Desirability	3.68 ± 0.15	3.80 ± 0.14	0.64	39	0.529
Intensity	4.18 ± 0.16	4.12 ± 0.14	0.29	39	0.775
Pill-using women, core sample					
Pleasantness	4.08 ± 0.18	4.25 ± 0.20	0.67	30	0.510
Desirability	3.84 ± 0.23	3.69 ± 0.21	0.58	30	0.567
Intensity	3.95 ± 0.22	4.11 ± 0.19	0.60	30	0.554

Table S4. Mean scores given to individual male odour donors when odours were presented to MHC-similar or MHC-dissimilar women. All women were normally cycling (data are from Session 1). The upper set of scores relates to standardized scores to control for differential use of the rating scale by female raters, while the lower set shows raw scores. Within each set, the upper 3 rows relate to all ratings, the lower 3 is restricted to ratings from the core sample of women (from UK participants and odours that were not confounded by deodorants or tobacco smoke). N=79 and 52 (the numbers of men who were scored in both the similar and dissimilar conditions).

Measure	Mean ± s.e.		Paired *t*	*df*	*p*
	Similar	Dissimilar			
Standardised scores, All ratings					
Pleasant	-0.0018 ± 0.08	0.0477 ± 0.08	-0.54	78	0.588
Intensity	-0.0450 ± 0.07	-0.0100 ± 0.07	-0.35	78	0.724
Desirability	-0.0417 ± 0.07	0.0449 ± 0.07	-0.93	78	0.353
Standardised scores, Core sample (UK women, no confounds)					
Pleasant	-0.0632 ± 0.09	0.0679 ± 0.09	-1.06	51	0.294
Intensity	-0.0154 ± 0.10	0.0292 ± 0.09	-0.38	51	0.703
Desirability	-0.0238 ± 0.09	0.0135 ± 0.09	-0.27	51	0.788
Raw scores, All ratings					
Pleasant	3.97 ± 0.13	4.06 ± 0.12	-0.63	78	0.531
Intensity	4.31 ± 0.13	4.27 ± 0.13	0.27	78	0.789
Desirability	3.41 ± 0.13	3.59 ± 0.13	-1.22	78	0.226
Raw scores, Core sample (UK women, no confounds)					
Pleasant	3.74 ± 0.13	3.94 ± 0.13	-1.37	51	0.177
Intensity	4.21 ± 0.17	4.19 ± 0.15	-0.11	51	0.915
Desirability	3.24 ± 0.16	3.45 ± 0.15	-1.05	51	0.298

Table S5. Odour ratings and male heterozygosity. Mean scores and independent-samples t tests between men who were heterozygous at all 3 MHC loci and those who were homozygous for one of more loci. No comparisons were significantly different.

Session	Measure	Zygosity	N	Mean	s.e.	t	df	p
First	***All ratings***							
	Pleasantness	Het	73	0.007	0.06	0.20	93	0.838
		Hom	22	-0.018	0.11			
	Intensity	Het	73	-0.023	0.05	-1.20	93	0.232
		Hom	22	0.111	0.10			
	Desirability	Het	73	0.011	0.06	0.17	93	0.869
		Hom	22	-0.009	0.11			
	Core sample							
	Pleasantness	Het	61	0.027	0.07	0.65	79	0.520
		Hom	20	-0.064	0.14			
	Intensity	Het	61	-0.005	0.06	-1.36	79	0.177
		Hom	20	0.177	0.13			
	Desirability	Het	61	0.035	0.07	0.76	79	0.451
		Hom	20	-0.072	0.14			
Second	***Non-users: all ratings***							
	Pleasantness	Het	70	0.043	0.07	1.57	89	0.121
		Hom	21	-0.208	0.18			
	Intensity	Het	70	0.061	0.08	-1.09	89	0.278
		Hom	21	0.237	0.14			
	Desirability	Het	70	0.044	0.06	1.62	89	0.110
		Hom	21	-0.192	0.17			
	Non-users: core sample							
	Pleasantness	Het	58	0.007	0.08	0.87	76	0.386
		Hom	20	-0.149	0.19			
	Intensity	Het	58	-0.012	0.09	-0.99	76	0.326
		Hom	20	0.166	0.17			
	Desirability	Het	58	0.033	0.08	0.76	76	0.450
		Hom	20	-0.097	0.19			
	Pill users: all ratings							
	Pleasantness	Het	66	0.087	0.09	1.01	80	0.315
		Hom	16	-0.104	0.15			
	Intensity	Het	66	0.077	0.08	1.55	80	0.125
		Hom	16	-0.201	0.17			
	Desirability	Het	66	0.063	0.09	1.12	80	0.267
		Hom	16	-0.151	0.15			
	Pill users: core sample							
	Pleasantness	Het	47	-0.108	0.10	-1.00	58	0.322
		Hom	13	0.115	0.21			
	Intensity	Het	47	-0.112	0.10	0.62	58	0.536
		Hom	13	-0.253	0.21			
	Desirability	Het	47	-0.056	0.10	-0.77	58	0.446
		Hom	13	0.108	0.20			

Table S6. Frequency of women's memory associations between the odours of partners (current or previous) or relatives and either MHC-similar or dissimilar odours presented in the experiment. Frequencies are tested using two-tailed Fisher's exact tests (after Wedekind et al. 1995).

Sample	Session	Association	MHC-similar	MHC-dissimilar	p
All	1	(Ex-)Partner	28/331	30/329	0.785
		Relative	13/331	19/329	0.283
Core	1	(Ex-)Partner	16/229	14/217	0.852
		Relative	8/229	14/217	0.190
All, non-users	2	(Ex-)Partner	15/175	12/179	0.552
		Relative	6/175	6/179	1.0
All, pill-users	2	(Ex-)Partner	7/121	11/118	0.335
		Relative	3/121	7/118	0.212
Core, non-users	2	(Ex-)Partner	11/127	8/129	0.484
		Relative	5/127	2/129	0.279
Core, pill-users	2	(Ex-)Partner	4/88	8/86	0.246
		Relative	1/88	6/86	0.063

Figure S1. Repeatability of scoring for odour pleasantness

Histograms are frequency distributions of Spearman rank correlation coefficients calculated for scores awarded to six shirts by individual raters, either within test sessions (inter-session interval: approximately 1h) or between test sessions (inter-session interval: approximately 3 months). Scores are repeatable for each comparison except for pill users between sessions.

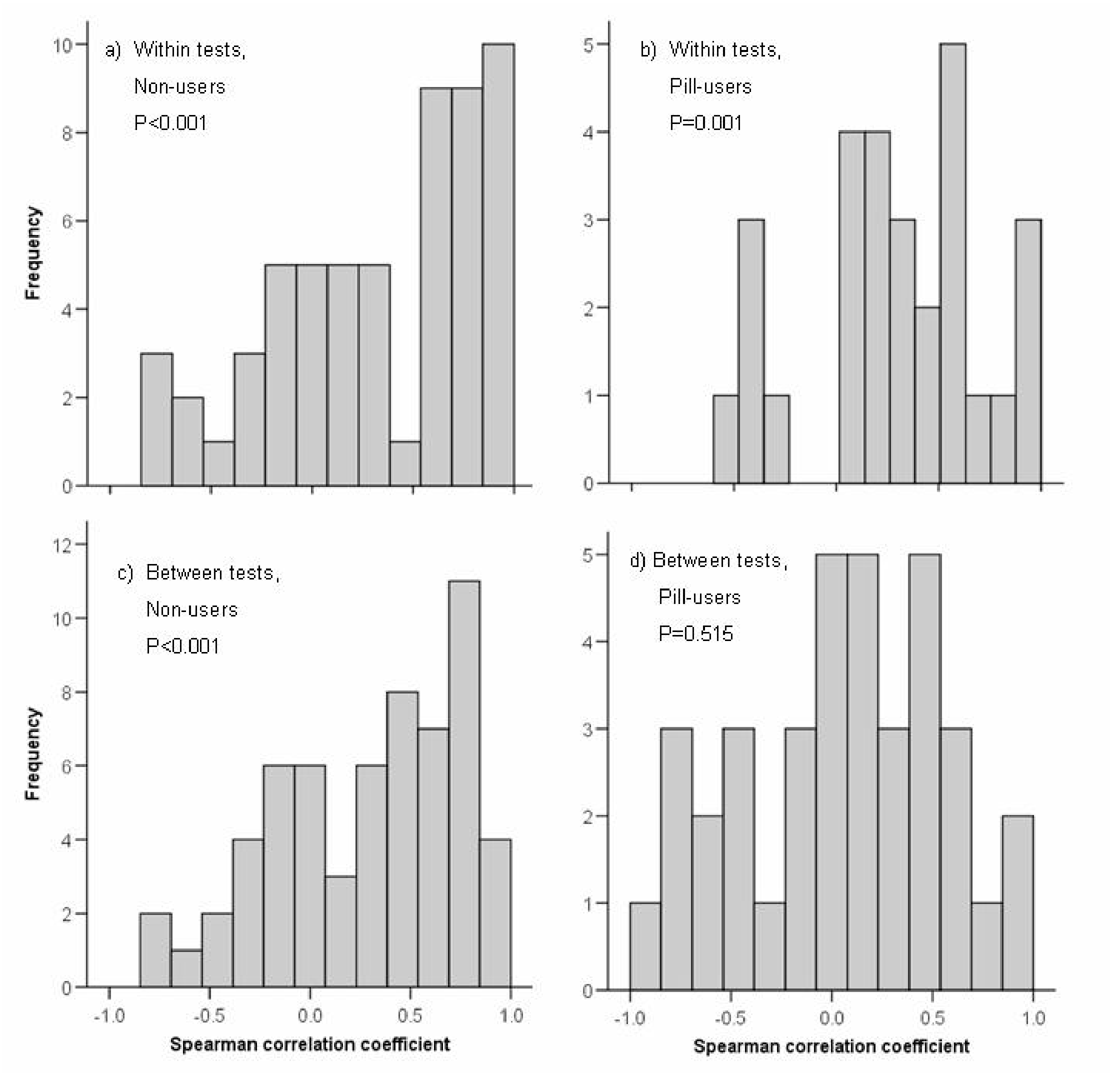

Figure S2. Repeatability of scoring for odour intensity and partnership desirability.

Histograms are frequency distributions of Spearman rank correlation coefficients calculated for scores awarded to six shirts by individual raters, either within test sessions (inter-session interval: approximately 1h) or between sessions (inter-session interval: approximately 3 months). Left-hand column= non-users; right-hand column= pill-users. Statistics are one-tailed *t*-tests against chance value of zero.

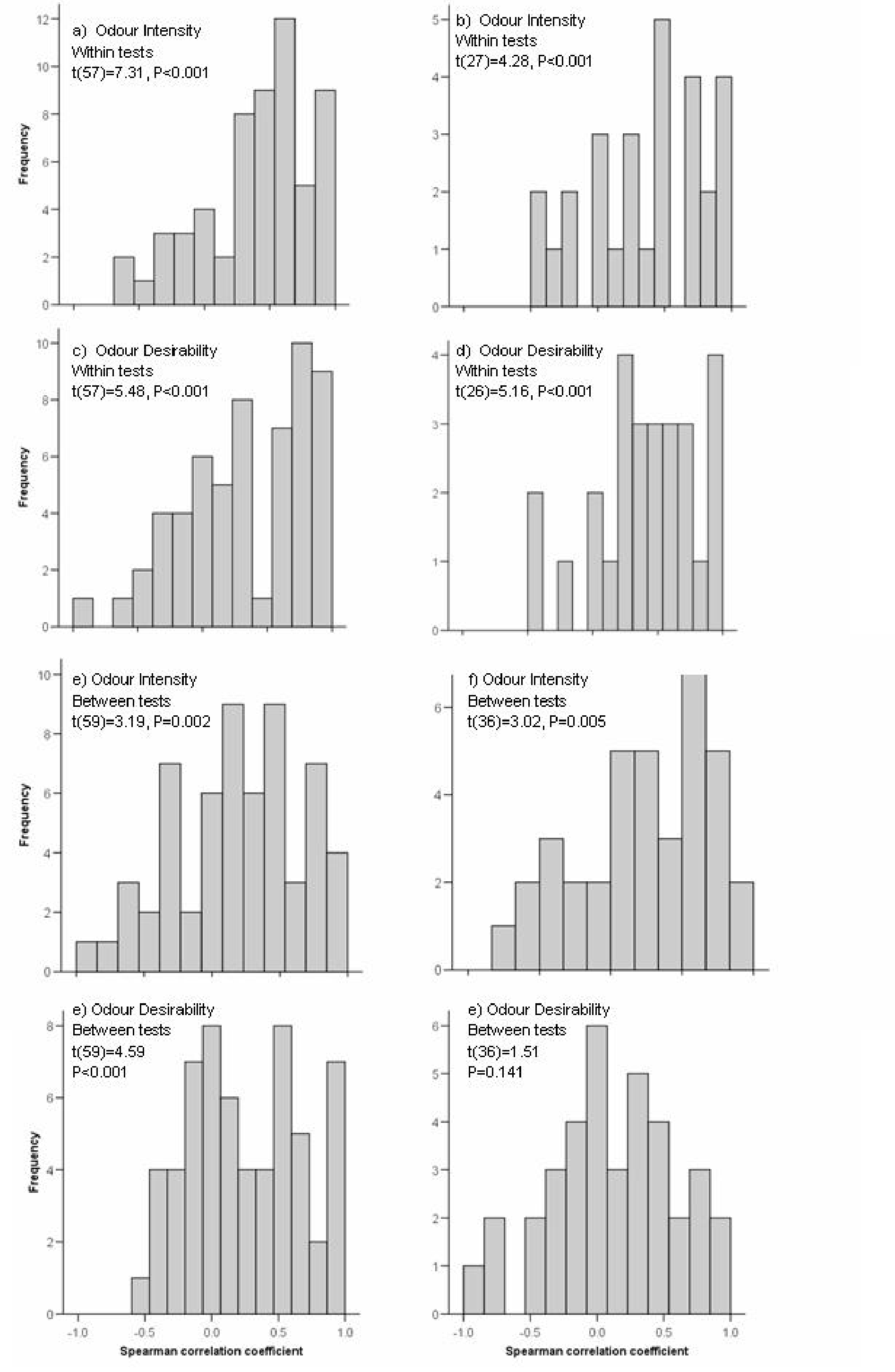

Supplement: Additional tables and figures — Methodological detail, repeatability of ratings and additional results [file rspb20080825s03.pdf]
